# Neurocognitive Rehabilitation in Parkinson's Disease with Motor Imagery: A Rehabilitative Experience in a Case Report

**DOI:** 10.1155/2015/670385

**Published:** 2015-01-06

**Authors:** Federico Zangrando, Giulia Piccinini, Andrea Pelliccioni, Vincenzo Maria Saraceni, Teresa Paolucci

**Affiliations:** Complex Operative Unit in Physical Medicine and Rehabilitation, Umberto I Hospital, “Sapienza” University of Rome, Piazzale Aldo Moro 5, 00185 Rome, Italy

## Abstract

A 50-year-old female with Parkinson's disease underwent a neurocognitive rehabilitation program consisting of one-hour-lasting sessions attended twice a week for three months. The balance and the risk of falls were determined using the Tinetti Balance and Gait Evaluation Scale. The pain was determined using the Visual Analog Scale and the course of the disease was examined using the Unified Parkinson's Disease Rating Scale (UPDRS). Endpoints were before the treatment, at the end of the treatment, and at a 12-week follow-up. The aim of this study is to evaluate the efficacy of neurocognitive rehabilitation in PD with motor imagery. Primary outcome is the improvement in balance and the falls risk reduction; secondary outcome is lower limb pain reduction.

## 1. Introduction

Balance and gait impairments are not fully addressed by pharmacological agents in Parkinson's disease (PD); thus nonpharmacological approaches are necessary. For patients with central nervous system (CNS) lesions and sensorimotor impairments, a solid introduction to motor imagery (MI) is crucial for optimizing the retraining of motor function [1]. The motor imagery is a biological phenomenon and results from cognitive processes closely related to our world experience. Through mental images situations and actions can be anticipated, formulating behavioral strategies to be adopted. The image acts as a bridge between perception and memory and between perception and motor control; furthermore the perception capacity is closely related to the perception of pain. PD patients usually present impairments in terms of motor control as well as in sensory integration, resulting in static and dynamic postural control deficits. Another symptom of the most common nonmotorsymptoms of Parkinson's disease is chronic pain; we hypothesize that MI could be an effective instrument against pain, restoring the somesthetic channel suppressed and thus reestablishing the coherency of afference to the central level (CNS). This study was performed to confirm the validity of neurocognitive rehabilitation [2] in PD with MI to improve balance and reduce lower limb pain, based on the hypothesis that balance control is impaired due to disorders in movement, motor planning, perception [3], and cognitive processes [4].

## 2. Case Presentation

A 50-year-old female patient who had Parkinson's disease for over 12 years and had never undergone rehabilitation was admitted to the physical medicine and rehabilitation outpatient unit. In 2002, as a 38-year-old, right handed person, she noticed a loss of fine movements in the fingers and left tremor finger, with pain and weakness in the lower limbs. In 2005, her movement began to slow, and bradykinesia developed on the right side. By 2002 to 2003, her Parkinsonian signs and symptoms were well controlled by amantadine (300 mg/day). In 2004, due to greater deficits in ambulation, which was possible only with the aid of a walking stick, she switched therapy to pergolide mesylate (3 mg/day), which was halted in 2005 for mitral flutter. Then, she began therapy with levodopa (400 mg/day). In 2010, there was an increase in bradykinesia, tremors, and ON-OFF moments, prompting amendment of her therapeutic regimen with levodopa (400 mg/day) and ropinirole (3 mg/day). On admission to a rehabilitation outpatient unit, she was receiving ropinirole (3 mg/day), levodopa (400 mg/day), carbidopa (93.75 mg/day), entacapone (600 mg/day), and levothyroxine (200 micrograms/day). The symptoms of the patient were well managed by the pharmacological therapy and she usually had no more than one “OFF” episode per day, defined as a state of impaired motor function in which the patient responds poorly to levodopa. It alternates with periods of improved mobility, “ON” phases, during which the patient responds to the drug.

Her minimental status examination (MMSE) score was normal (26/30), as was the cranial magnetic resonance imaging. A neurological examination showed no evidence of deficits in the cranial nerves, superficial or deep sensitivity, or cerebellar activity. Her bilateral symptoms include an increase in freezing of gait, festination, bradykinesia, and dyskinetic movements of the trunk and retropulsion and rigidity in all 4 limbs. At this point, the patient discontinued working.

Ethics approval from the human studies committee of Policlinico Umberto I Hospital, “Sapienza” University of Rome, was obtained and the participant provided informed written consent, according to the Declaration of Helsinki.

**Figure 1:**
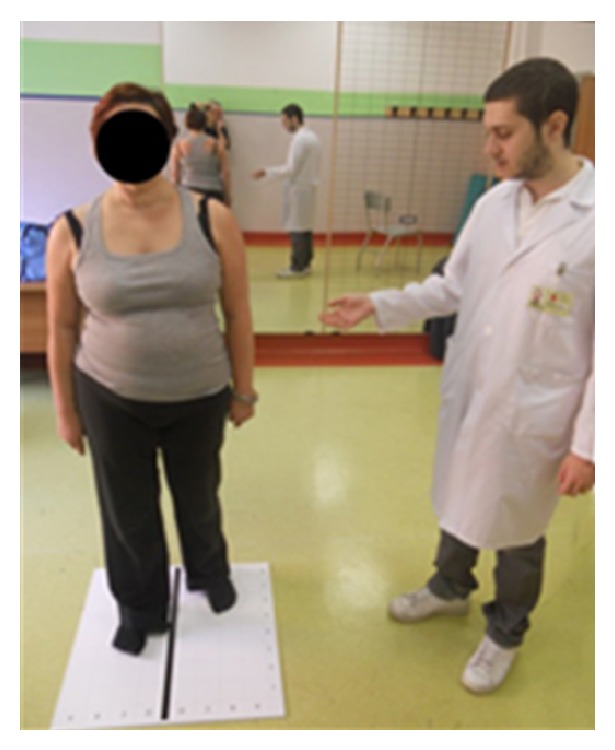
Exercise for the stride phase using a checkered board.

**Figure 2:**
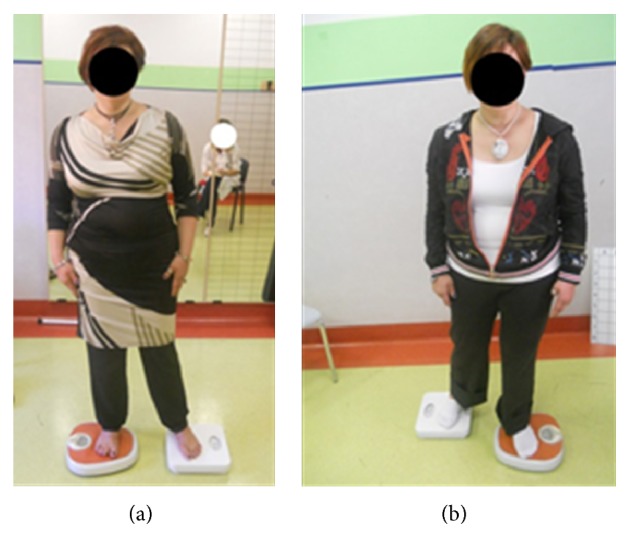
Exercise for the stance phase using personal weighing machines.

We proposed a 3-month neurocognitive rehabilitation program with the use of MI comprising 20 sessions (1 hour each, twice per week). Motor imagery exercises have been administered as a neurocognitive rehabilitation regimen, linking perception and movement, to develop strategies for solving the task in the exercise. The movement in the exercise can thus only be performed if the patient can evoke accurate motor imagery of the required movement, allowing her to self-correct [5]. When the patient uses motor imagery correctly, her behavior should be modified. Our patient had to imagine the following actions before performing them: transitioning from a seated position to a standing position; stride (Figure 1) and stance phase (Figure 2); start of gait; and gait on different paths and grounds. After performing the required movement, the patient had to compare what she had imagined with what she perceived to identify and correct the possible errors by herself more easily.

**Figure 3:**
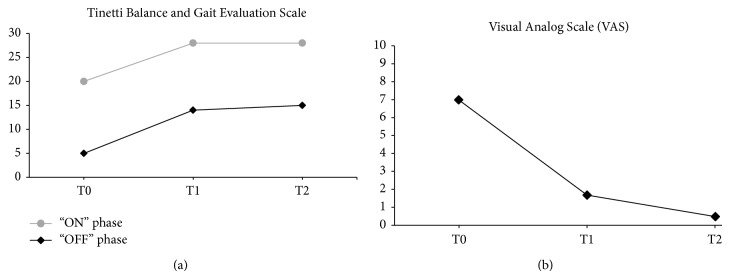
Graphic representation of Tinetti (a) and VAS (b) results.

**Figure 4:**
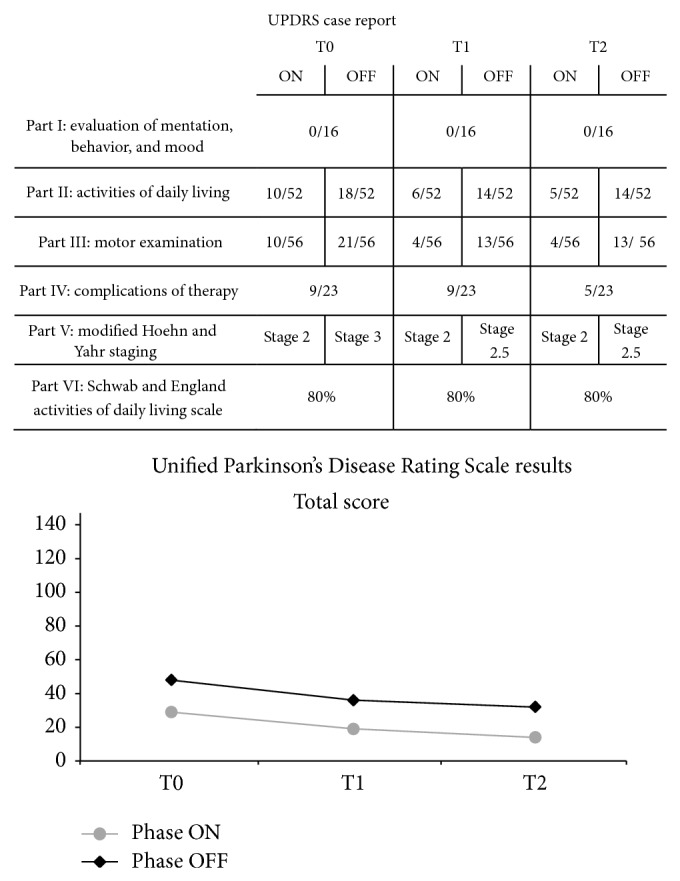
Unified Parkinson's Disease Rating Scale results with graphic representation of total score.

Data were collected by a blinded tester specialist in physical medicine and rehabilitation at the beginning of treatment (T0), at the end of treatment (3 months) (T1), and at the 3-month follow-up (T2). We analyzed the balance and the risk of falls using the Tinetti Balance and Gait Evaluation Scale (28-point scale: <19 indicates a high risk for falls; 19–24 indicates a moderate risk for falls). The Visual Analog Scale (VAS) (scored 0–10) was used to quantify the pain subjectively. The course of the disease was examined using the Unified Parkinson's Disease Rating Scale (UPDRS). The Tinetti scale identified improvements in balance and indicated that the patient decreased her risk of falling, both in “ON” and “OFF” phases; these effects persisted at T2 (Figure 3). According to these results, the Pull Test, performed in the “ON” and “OFF” phases, showed an improvement in balance from T0 to T1 with confirmed results at the follow-up (“ON” phase: 1 (T0), 0 (T1), 0 (T2); “OFF” phase: 2 (T0), 1 (T1), 1 (T2)). The VAS, decreasing from 7 (T0) to 1.7 (T1), confirmed the decline in lower limb pain as a freezing prodrome, improving to 0.5 at T2. The UPDRS showed improved motor activity in the “ON” and “OFF” phases, with a resulting improvement in the activities of daily living. This score remained constant at the follow-up (Figure 4).

## 3. Discussion

Our research was performed to confirm the validity of neurocognitive rehabilitation in PD with MI to improve balance and lower limb pain reduction. The use of MI within the neurocognitive rehabilitation, resulted to be effective in improving postural stability acting as a bridge between perception and motor control [6, 7] as frontoparietal regions, basal ganglia, and medial cerebellum are involved during MI of dynamic balance, since the enhancing effect of MI on cortical excitability and recruitment patterns depend on imagery quality [8, 9]. Pain perception is altered in PD, such as elevations in sensory threshold, in which the interaction between sensory input and motor output modulates [10]. In particular, lower limb pain is a variant of central pain and merits recognition as a specific nonmotor phenotype in PD. Thus MI in rehabilitation training maybe modifies pain memory. Mental rehearsal of the subnociceptive images was found to modulate the perception of the nociceptive sensation felt prior to the imagery. The significant improvement in the VAS scale can be explained by the assumption that the pain we examined is related to an alteration in CNS, corresponding to an alteration in perception or rather in loss of the ability to integrate different sensory information [11]. Thus, pain can be considered as the product of the output of a widely distributed neural network in the brain rather than directly by sensory input evoked by an injury [12, 13]. Rehabilitation with motor imagery (MI) was therefore proposed to bring back coherence between afferences at central level, which is needed to rebuild the body self and relieve the pain [14]. The results of a study on functional magnetic resonance imaging (fMRI) have shown how using the motor imagery within the neurocognitive rehabilitative approach can induce changes in the perception of pain by changing the memory of pain at central level [15].

## 4. Conclusion

Based on the results of our study, the hypothesis that the presence of motor and perceptive disorders underlines the balance impairments in people with PD is confirmed, as is the validity of motor imagery as an instrument to elaborate the somesthetic information that is necessary to execute the action more accurately. We conclude by affirming that neurocognitive rehabilitation based on MI can be considered an effective rehabilitative approach in PD patients.
